# Impact of Day-3 embryo cell number on pregnancy, obstetric and perinatal outcomes in frozen-thawed single blastocyst transfer cycles

**DOI:** 10.3389/fendo.2026.1777070

**Published:** 2026-06-22

**Authors:** Feng Xiong, Caiyun Wan, Xiaoqi Peng, Qing Sun, Shiru Xu, Lianghui Diao, Peilin Chen, Huixian Zhong, Meilan Mo, Tao Zhang

**Affiliations:** 1 Department of Obstetrics and Gynaecology, Faculty of Medicine, The Chinese University of Hong Kong, Hong Kong, Hong Kong SAR, China; 2 Reproductive Medicine Center, Shenzhen Zhongshan Obstetrics & Gynecology Hospital, Shenzhen, China; 3 Shenzhen Key Laboratory of Reproductive Immunology for Peri-implantation, Guangdong Engineering Technology Research Center of Reproductive Immunology for Peri-implantation, Shenzhen Zhongshan Institute for Reproductive Medicine and Genetics, Shenzhen Zhongshan Obstetrics & Gynecology Hospital, Shenzhen, China

**Keywords:** Day-3 cell number, live birth, obstetric outcomes, perinatal outcomes, single blastocyst transfer

## Abstract

**Objective:**

To evaluate the impact of Day-3 embryo cell number on pregnancy, obstetric, and perinatal outcomes after frozen-thawed single blastocyst transfer (SBT).

**Methods:**

This retrospective cohort study included 6437 frozen–thawed SBT cycles performed between 2016 and 2024 at a large reproductive center. Cycles were stratified by Day-3 cell number into six groups (≤ 5, 6, 7, 8, 9, and ≥ 10 cells). Pregnancy outcomes included clinical pregnancy, ongoing pregnancy, live birth, and early miscarriage rates. Obstetric and perinatal outcomes were assessed in 3191 singleton live-birth cycles. Multivariable logistic regression, stratified sensitivity analyses, and propensity score matching (PSM) were performed to adjust for relevant covariates.

**Results:**

Compared with the 8-cell group, blastocysts derived from Day-3 embryos with ≤5 or 6 cells were associated with significantly lower odds of live birth (≤5-cell: aOR 0.69, 95% CI 0.56–0.85; 6-cell: aOR 0.76, 95% CI 0.60–0.95), clinical pregnancy, and ongoing pregnancy after multivariable adjustment. These associations were further confirmed after PSM. In stratified analyses, the adverse associations observed for the ≤5-cell group remained consistent across subgroups defined by blastocyst quality or blastocyst day. In contrast, the associations observed in the 6-cell group remained directionally similar to those in the overall analysis, although they were no longer statistically significant among Day-6 blastocysts. Regarding obstetric and perinatal outcomes, neither the ≤5-cell nor the 6-cell group showed significant differences in gestational age, preterm birth, cesarean delivery, major obstetric complications, neonatal sex, birth weight, or birth defects compared with the 8-cell group.

**Conclusions:**

Overall, blastocysts derived from Day-3 embryos with ≤5 or 6 cells were associated with reduced pregnancy outcomes after frozen-thawed SBT. However, among singleton live births, no adverse associations with obstetric or perinatal outcomes were observed in either group. These findings may help inform blastocyst selection and patient counselling during IVF treatment.

## 1 Introduction

The *in vitro* fertilization (IVF) procedure became the primary treatment for infertility worldwide following the successful birth of the first IVF baby in 1978. During those early decades, embryo transfer was mainly performed at the cleavage stage, and the embryos were evaluated based on morphological features such as blastomere number, degree of fragmentation, and cell symmetry (1). The blastomere number or cell number—the number of cells formed during cleavage divisions of the fertilized oocyte—reflects the developmental rate, and it is widely considered a key indicator of embryo quality (2). According to most studies, the ideal cell number for Day-3 embryos is 7 to 9 cells, with 8 being optimal for embryo transfer due to its association with higher implantation and pregnancy rates (1, 3). In contrast, embryos exhibiting delayed or accelerated cleavage are generally considered to have reduced developmental competence (4). Previous studies have also reported associations between abnormal cleavage dynamics and an increased risk of chromosomal abnormalities (5, 6), although Day-3 morphological features alone cannot reliably determine embryo chromosomal status.

In the 1990s, embryo culture was extended to the blastocyst stage following the introduction of sequential culture media. Because blastulation is itself indicative of developmental competence, the ability of a cleavage-stage embryo to achieve the blastocyst stage during culture is considered a robust tool for embryo selection (7). Indeed, blastocyst transfer is now commonly used in both IVF and intracytoplasmic sperm injection (ICSI) cycles, and superior clinical outcomes have been achieved compared to cleavage-stage transfer (8, 9). Consequently, blastocysts derived from Day-3 embryos with different cell numbers are inevitably encountered in clinical practice and may be selected for transfer. This raises two clinically relevant questions: (1) Does deviation from the optimal 8-cell stage on day 3 compromise pregnancy success despite subsequent development to the blastocyst stage? and (2) Does the transfer of blastocysts derived from embryos that exhibited suboptimal development at the cleavage-stage increase the risks of adverse obstetric and perinatal outcomes?

The relationship between Day-3 cell number and pregnancy outcomes in single blastocyst transfer (SBT) cycles has already been investigated in several notable studies, although the findings are inconsistent. Specifically, Zhao et al. reported significantly lower live birth rates in the ≤ 6-cell group, compared with the 8-cell group, in frozen-thawed embryo transfer (FET) cycles (10). Conversely, Li et al. reported that there were no significant differences in clinical pregnancy and implantation rates between the ≤6-cell group and either the 7–9-cell or ≥10-cell groups in FET cycles (11). Furthermore, several other studies have also reported conflicting results (12, 13). Importantly, these studies have focused almost exclusively on pregnancy outcomes, with limited investigation into obstetric and perinatal outcomes, which are equally critical to families.

Therefore, the present study aims to comprehensively evaluate the impact of Day-3 cell number on pregnancy, obstetric, and perinatal outcomes in frozen-thawed SBT cycles. Our findings are expected to provide evidence to support patient counselling for SBT cycles involving blastocysts derived from embryos with atypical cleavage rates and to inform embryo selection strategies.

## 2 Materials and methods

### 2.1 Study design and patients

**Figure 1:**
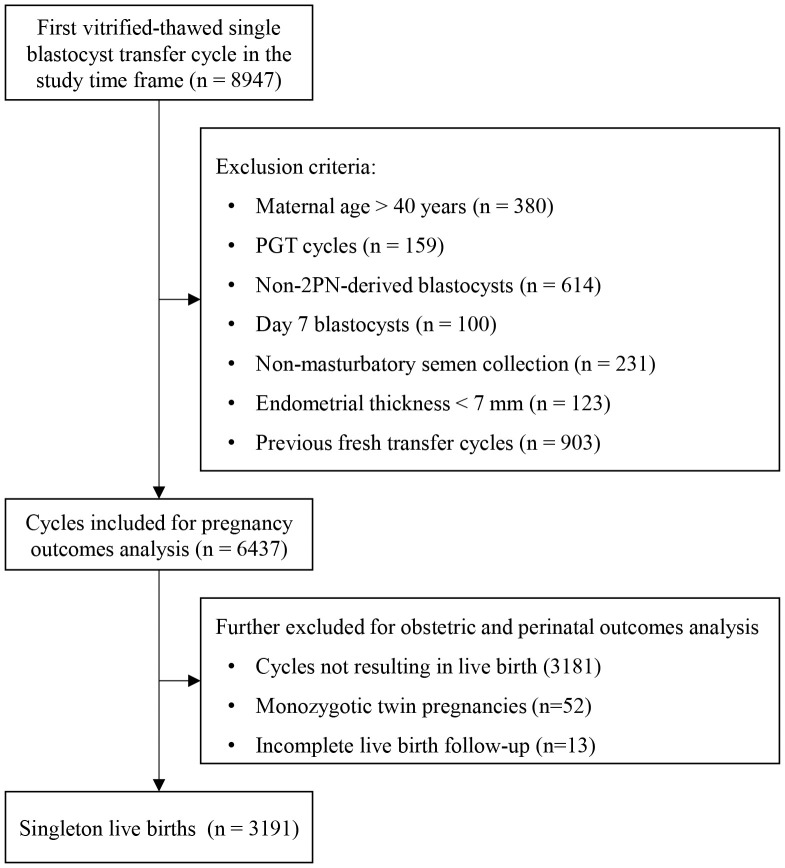
Flowchart of study population selection.

This retrospective study included patients who underwent frozen-thawed SBT at our center between 2016 and 2024. All data was retrieved from the electronic medical records. To minimize potential bias, only the first embryo transfer cycle for each patient was included. All transferred blastocysts were derived from oocytes with two pronuclei (2PN) following insemination with ejaculated sperm. The exclusion criteria included maternal age > 40 years at transfer, Day-7 blastocyst transfer, endometrial thickness < 7 mm on the day of progesterone initiation, and biopsy cycles. In total, 6437 eligible frozen-thawed SBT cycles were included in the final analysis. A flowchart of patients recruitment is shown in **Figure 1**. For the analysis of obstetric and perinatal outcomes, 52 monozygotic twin pregnancies were excluded. In addition, 13 cycles with incomplete live-birth follow-up were also excluded, including 8 cycles with missing live-birth outcomes and 5 cycles in which the patient reported a live birth but declined to provide additional outcome information.

This study was approved by the Research Ethics Committee of Shenzhen Zhongshan Obstetrics & Gynecology Hospital (SZZSECHU-F-2025074). All patients had provided consent for IVF treatment procedures. Because this study retrospectively used anonymized data from electronic medical records, the requirement for additional informed consent for this retrospective analysis was waived by the ethics committee.

### 2.2 Ovarian stimulation and embryo culture

Ovarian stimulation protocols were determined by IVF clinicians according to individual ovarian reserve and anticipated response. The main strategies included the long gonadotrophin-releasing hormone (GnRH) agonist protocol; the GnRH antagonist protocol; and the clomiphene-based mild stimulation protocol. Follicular development was monitored by serial transvaginal ultrasonography, and final oocyte maturation was triggered with hCG and/or GnRH agonist when at least two follicles reached a diameter ≥ 18 mm. Oocyte retrieval was performed 36–37 hours post-trigger under transvaginal ultrasound guidance. Ejaculated semen samples were processed by density gradient centrifugation. Fertilization was achieved using either conventional IVF methods or ICSI, depending on semen parameters. Zygotes were cultured in sequential media (Quinn’s Advantage Cleavage Medium followed by Quinn’s Advantage Blastocyst Medium, SAGE, USA), with medium renewal on Day 3. Each embryo was individually cultured in a 25 μL droplet. Embryo development was assessed at standardized time points according to the Istanbul consensus (1). Day-3 cleavage-stage embryos were assessed at 68 ± 1 hours after insemination, with the expected developmental stage being the 8-cell stage. Blastocyst quality was graded according to the Gardner and Schoolcraft scoring system (14). Blastocysts were classified as high quality (AA, AB, BA, or BB), or low quality (AC, CA, BC, or CB). Blastocysts with both the inner cell mass and trophectoderm graded as C were considered to be of extremely poor quality and were discarded.

When multiple blastocysts were available for the same patient, the blastocyst with the better morphological grade was prioritized for transfer. If blastocysts had comparable morphological quality, Day-5 blastocysts were generally prioritized over Day-6 blastocysts. Day-3 cell number was not used as a primary selection criterion and was considered only when blastocysts were comparable in both morphological grade and developmental day.

### 2.3 Blastocyst vitrification and warming

Fully expanded blastocysts observed on Day 5 or Day 6 after insemination underwent artificial shrinkage by laser prior to vitrification. Vitrification and subsequent warming were performed using commercial kits (KITAZATO, Shizuoka, Japan) according to the manufacturer’s instructions. Warming was performed 3–4 hours before transfer. Immediately after warming, frozen-thawed blastocysts underwent laser-assisted hatching, as previously described (15).

### 2.4 Endometrial preparation and luteal support

Endometrial preparation for FET cycles included natural cycle (NC), hormone replacement treatment (HRT) cycle, and ovulation induction (OI) protocols. The choice of protocol was determined by clinicians according to patients’ menstrual and ovulatory characteristics. For patients with regular ovulation, an NC protocol was used. Follicular monitoring began on days 8–10 of the menstrual cycle using transvaginal ultrasonography. Ovulation timing was determined based on follicular development or the urinary LH surge. When the leading follicle reached ≥18 mm or a urinary LH surge was detected, ovulation was allowed to occur spontaneously or was triggered using 10000 IU hCG according to clinical judgement. Oral dydrogesterone 20 mg twice daily was administered for 5 days before blastocyst transfer. For patients with ovulatory dysfunction, an HRT protocol was used. Endometrial preparation began on days 2–3 of the menstrual cycle with oral estradiol at 4 mg/day. The dose was increased by 2 mg every 5 days. Intramuscular progesterone 60 mg daily was then administered for 6 days before blastocyst transfer. For patients considered unsuitable for NC or HRT because of poor follicular or endometrial development, an OI protocol was used. Endometrial preparation began with oral letrozole from days 3–5 of the menstrual cycle for 5 consecutive days. From day 10 of the menstrual cycle, human menopausal gonadotropin was administered according to follicular response. When the dominant follicle reached 18 mm in diameter, hCG was administered intramuscularly. Ovulation was confirmed by ultrasound 36 hours later. After ovulation was confirmed, subsequent treatment was the same as that used in NC cycles.

The interval from SBT to progesterone initiation was uniform within each endometrial preparation protocol regardless of whether the blastocyst had been vitrified on Day 5 or Day 6. On the day of embryo transfer, luteal-phase support in all protocols was changed to oral dydrogesterone 20 mg twice daily and vaginal progesterone gel 90 mg daily. Serum hCG was measured 11 days after blastocyst transfer. If pregnancy was achieved, luteal support was continued until 12 weeks of gestation.

### 2.5 Outcome evaluation

Pregnancy, obstetric, and perinatal outcomes were assessed according to previous definitions (16). Clinical pregnancy was defined as an intrauterine gestational sac under transvaginal ultrasound approximately 30 days after SBT. Ongoing pregnancy was defined as the pregnancy continuing beyond 12 weeks of gestation, whereas early miscarriage refers to spontaneous termination of an intrauterine pregnancy before 12 weeks of gestation. Live birth rate was calculated as the proportion of transfers resulting in at least one live-born infant. Gestational age was calculated from the date of SBT by adding 19 days. Preterm birth was defined as delivery before 37 completed weeks of gestation, and extremely preterm birth was defined as delivery before 34 completed weeks of gestation. GDM was diagnosed using a 75-g oral glucose tolerance test at 24–28 weeks of gestation. GH was defined as blood pressure ≥140/90 mmHg after 20 weeks of gestation in previously normotensive women. PPH was defined as blood loss ≥500 mL after delivery. Birth defects were classified according to the International Statistical Classification of Diseases and Related Health Problems, 10th Revision. Neonatal incubator admission was defined as any postnatal admission to a neonatal incubator.

### 2.6 Statistical analysis

All analyses were conducted using R (version 4.4.4; R Foundation for Statistical Computing, Vienna, Austria) and SPSS software (version 22.0; IBM Corp., Armonk, NY, USA). Continuous variables are presented as medians with interquartile ranges (IQRs) and were compared using the Kruskal–Wallis test. Categorical variables are expressed as frequencies and percentages (n, %), and these were compared using chi-square test or Fisher’s exact test, as appropriate. All pairwise comparisons with the 8-cell group were adjusted for multiple testing using Bonferroni correction, with an adjusted significance threshold of α = 0.01 (0.05/5). Standardized mean differences (SMDs) were additionally calculated to assess baseline imbalance between each Day-3 cell-number group and the 8-cell reference group. For continuous variables, SMDs were calculated as the difference in means divided by the pooled standard deviation; for categorical variables, level-specific SMDs were calculated as standardized differences in proportions.

Multivariable logistic regression was used to assess the associations between Day-3 cell number and pregnancy outcomes, with adjustment for the following covariates: maternal and paternal age at oocyte pick-up, body mass index (BMI), anti-Müllerian hormone (AMH), infertility diagnosis, endometrial thickness, number of retrieved oocytes, Day-3 fragmentation and symmetry, blastocyst day and quality, storage duration, insemination method, endometrial preparation protocol, parity, and gravidity. Because blastocyst day and quality may partly lie on the developmental pathway between Day-3 cell number and pregnancy outcomes, models adjusted for these variables were interpreted as estimating associations conditional on subsequent blastocyst development and morphology, rather than the total effect of cell number across all Day-3 cleavage-stage embryos. Adjusted odds ratios (aORs) with 95% confidence intervals (CIs) were reported.

Sensitivity analyses were performed among high-quality blastocysts, low-quality blastocysts, Day-5 blastocysts, and Day-6 blastocysts to assess the consistency of the associations between Day-3 cell number and pregnancy outcomes. Multivariable logistic regression was performed within each subgroup using the 8-cell group as the reference, with adjustment for the same covariates as in the main analysis, excluding the stratification variable where appropriate. As an additional sensitivity analysis, propensity score matching (PSM) was performed for each group versus the 8-cell reference group. Propensity scores were estimated using the same covariates as in the main analysis. One-to-one nearest-neighbor matching was applied without replacement, using a caliper of 0.2. Covariate balance before and after matching was assessed using SMDs, and pregnancy outcomes after matching were compared using weighted logistic regression models accounting for matching weights and subclasses.

All statistical tests were two-sided, and a *P* value < 0.05 was used to assess statistical significance except for multiple comparisons, where Bonferroni-adjusted thresholds were applied.

## 3 Results

### 3.1 Baseline demographics and cycle characteristics

In total, 6437 frozen-thawed SBT cycles were included in the final analysis. The baseline demographic and cycle characteristics are summarized in **Table 1**. Compared with the 8-cell group, maternal and paternal ages were higher in the ≤ 5-, 6-, and 7-cell groups. AMH levels were lower in the ≤5-, 6-, 7-, and ≥10-cell groups, whereas the number of oocytes retrieved was lower in all other groups. Infertility duration, BMI, gravidity, and endometrial thickness were similar across groups. Prior parity was more frequent in the 7-cell group. Day-3 fragmentation degree differed significantly in the ≤5-, 6-, 7-, and 9-cell groups and Day-3 symmetry differed significantly in the ≤5-, 6-, 7-, 9- and ≥10-cell groups. The proportion of Day-5 blastocysts transferred was lower in the ≤5-, 6-, 7-, and 9-cell groups, and the proportion of high-quality blastocysts transferred was significantly lower in the ≤5-, 6-, 7-, 9- and ≥10-cell groups. Storage duration was longer in the ≤5- and 6-cell groups. Endometrial preparation protocols differed significantly only in the ≤5-cell group.

**Table 1:** Cycle characteristics and baseline demographics.

Variable	≤5-cell (N = 561)	6-cell (N = 412)	7-cell (N = 996)	8-cell (N = 3696)	9-cell (N = 373)	≥10-cell (N = 399)	P value
Maternal age at ET (years)	33.00 (30.00–36.00)*	33.00 (30.00–35.50)*	32.00 (30.00–35.00)*	32.00 (29.00–34.00)	32.00 (29.00–35.00)	32.00 (29.00–35.00)	<0.001
Paternal age at ET (years)	34.00 (31.00–38.00)*	34.00 (31.00–38.00)*	34.00 (31.00–37.00)*	33.00 (31.00–36.00)	33.00 (31.00–36.00)	33.00 (31.00–37.00)	<0.001
Maternal age at OPU (years)	32.00 (30.00–36.00)*	32.00 (30.00–35.00)*	32.00 (29.00–35.00)*	31.00 (29.00–34.00)	32.00 (29.00–34.00)	32.00 (29.00–35.00)	<0.001
Paternal age at OPU (years)	34.00 (31.00–37.00)*	34.00 (31.00–37.00)*	34.00 (31.00–37.00)*	33.00 (30.00–36.00)	33.00 (30.00–36.00)	33.00 (30.00–37.00)	<0.001
Infertility duration (years)	3.00 (1.50–4.00)	3.00 (2.00–4.00)	3.00 (2.00–4.00)	3.00 (1.00–4.00)	3.00 (1.00–4.00)	3.00 (2.00–4.00)	0.161
BMI (kg/m2)	21.09 (19.47–23.44)	21.43 (19.62–24.03)	21.10 (19.53–23.34)	21.22 (19.53–23.44)	20.82 (19.47–23.03)	21.62 (19.53–23.54)	0.288
AMH (ng/mL)	2.68 (1.41–4.41)*	3.10 (1.68–4.74)*	3.24 (1.92–5.25)*	4.31 (2.79–6.45)	4.23 (2.38–6.31)	3.79 (2.02–6.04)*	<0.001
Gravidity							0.006
0	301/561 (53.65)	223/412 (54.13)	537/996 (53.92)	2,124/3,696 (57.47)	220/373 (58.98)	255/399 (63.91)	
≥1	260/561 (46.35)	189/412 (45.87)	459/996 (46.08)	1,572/3,696 (42.53)	153/373 (41.02)	144/399 (36.09)	
Parity			*				0.012
0	455/561 (81.11)	330/412 (80.10)	795/996 (79.82)	3,103/3,696 (83.96)	311/373 (83.38)	340/399 (85.21)	
≥1	106/561 (18.89)	82/412 (19.90)	201/996 (20.18)	593/3,696 (16.04)	62/373 (16.62)	59/399 (14.79)	
Maternal infertility diagnosis	*		*				<0.001
Unexplained infertility	195/561 (34.76)	150/412 (36.41)	360/996 (36.14)	1,208/3,696 (32.68)	125/373 (33.51)	141/399 (35.34)	
Ovulation dysfunction	45/561 (8.02)	60/412 (14.56)	157/996 (15.76)	786/3,696 (21.27)	66/373 (17.69)	80/399 (20.05)	
Tubal factor	233/561 (41.53)	151/412 (36.65)	349/996 (35.04)	1,307/3,696 (35.36)	128/373 (34.32)	130/399 (32.58)	
Endometriosis	88/561 (15.69)	51/412 (12.38)	130/996 (13.05)	395/3,696 (10.69)	54/373 (14.48)	48/399 (12.03)	
Oocyte number	10.00 (6.00–16.00)*	12.00 (8.00–17.00)*	14.00 (8.00–20.00)*	17.00 (12.00–22.00)	15.00 (11.00–21.00)*	15.00 (9.00–21.00)*	<0.001
Insemination method	*	*	*				<0.001
ICSI	146/561 (26.02)	117/412 (28.40)	256/996 (25.70)	739/3,696 (19.99)	74/373 (19.84)	62/399 (15.54)	
IVF	415/561 (73.98)	295/412 (71.60)	740/996 (74.30)	2,957/3,696 (80.01)	299/373 (80.16)	337/399 (84.46)	
Day-3 fragmentation	*	*	*		*		<0.001
< 10%	326/561 (58.11)	259/412 (62.86)	719/996 (72.19)*	3,296/3,696 (89.18)	297/373 (79.62)*	365/399 (91.48)	
10–20%	152/561 (27.09)	110/412 (26.70)	243/996 (24.40)*	378/3,696 (10.23)	74/373 (19.84)*	33/399 (8.27)	
> 20%	83/561 (14.80)	43/412 (10.44)	34/996 (3.41)*	22/3,696 (0.60)	2/373 (0.54)*	1/399 (0.25)	
Day-3 symmetry	*	*	*		*	*	<0.001
Uneven	358/561 (63.81)	318/412 (77.18)	689/996 (69.18)	1,223/3,696 (33.09)	275/373 (73.73)	306/399 (76.69)	
Even	203/561 (36.19)	94/412 (22.82)	307/996 (30.82)	2,473/3,696 (66.91)	98/373 (26.27)	93/399 (23.31)	
Blastocyst day	*	*	*		*		<0.001
5	205/561 (36.54)	229/412 (55.58)	739/996 (74.20)	3,275/3,696 (88.61)	312/373 (83.65)	357/399 (89.47)	
6	356/561 (63.46)	183/412 (44.42)	257/996 (25.80)	421/3,696 (11.39)	61/373 (16.35)	42/399 (10.53)	
Storage days	80.00 (56.00–112.00)*	74.50 (50.00–107.00)*	69.00 (53.00–98.50)	66.00 (50.00–94.00)	68.00 (41.00–91.00)	64.00 (47.00–97.00)	<0.001
Endometrial thickness (mm)	9.00 (8.00–10.00)	9.00 (8.00–10.00)	9.00 (8.00–10.00)	9.00 (8.00–10.00)	9.00 (8.00–10.00)	9.00 (8.00–10.00)	0.584
Endometrial preparation	*						0.001
HRT	252/561 (44.92)	182/412 (44.17)	461/996 (46.29)	1,690/3,696 (45.73)	167/373 (44.77)	187/399 (46.87)	
NC	218/561 (38.86)	166/412 (40.29)	393/996 (39.46)	1,385/3,696 (37.47)	149/373 (39.95)	143/399 (35.84)	
OI	64/561 (11.41)	52/412 (12.62)	120/996 (12.05)	561/3,696 (15.18)	48/373 (12.87)	59/399 (14.79)	
Others	27/561 (4.81)	12/412 (2.91)	22/996 (2.21)	60/3,696 (1.62)	9/373 (2.41)	10/399 (2.51)	
Blastocyst quality	*	*	*		*	*	<0.001
High	300/561 (53.48)	243/412 (58.98)	767/996 (77.01)	3,447/3,696 (93.26)	304/373 (81.50)	330/399 (82.71)*	
Low	261/561 (46.52)	169/412 (41.02)	229/996 (22.99)	249/3,696 (6.74)	69/373 (18.50)	69/399 (17.29)*	

Values are presented as median (IQR) for continuous variables and n/N (%) for categorical variables.

Overall *P* values were calculated using the Kruskal–Wallis test for continuous variables and Pearson’s chi-square test or Fisher’s exact test for categorical variables.

*Significantly different from the 8-cell group at *P* < 0.01 using the Wilcoxon rank-sum test for continuous variables and the chi-square test or Fisher’s exact test for categorical variables. Bonferroni correction for five comparisons (0.05/5).

ET, embryo transfer; OPU, oocyte pick-up; BMI, body mass index; AMH, anti-Müllerian hormone; ICSI, intracytoplasmic sperm injection; IVF, *in vitro* fertilization; HRT, hormone replacement therapy; NC, natural cycle; OI, ovulation induction.

Standardized mean differences (SMDs) were additionally calculated to quantify baseline imbalance between each group and the 8-cell group (**Supplementary Table 1**). Overall, the SMD analysis showed consistent patterns with the pairwise comparisons in **Table 1**, with the most notable imbalances observed for blastocyst day, blastocyst quality, Day-3 fragmentation and symmetry, AMH level, and number of oocytes retrieved.

### 3.2 Pregnancy outcomes across Day-3 cell number groups

Pregnancy outcomes across the six groups are summarized in **Table 2**. Significant differences were observed among the groups in the rates of clinical pregnancy, ongoing pregnancy, live birth, and early miscarriage. Compared with the 8-cell group, blastocysts with ≤ 5, 6, or 7 cells on Day 3 were associated with significantly lower rates of clinical pregnancy, ongoing pregnancy, and live birth. Early miscarriage rates were also significantly higher in the ≤5-cell and 6-cell groups than in the 8-cell group.

**Table 2:** Pregnancy outcomes following frozen single blastocyst transfer stratified by Day-3 cell number.

Variable	≤5-cell (N = 561)	6-cell (N = 412)	7-cell (N = 996)	8-cell (N = 3696)	9-cell (N = 373)	≥10-cell (N = 399)	P value
Clinical pregnancy rate	265/561 (47.24)*	212/412 (51.46)*	601/996 (60.34)*	2,473/3,696 (66.91)	230/373 (61.66)	255/399 (63.91)	<0.001
Ongoing pregnancy rate	207/561 (36.90)*	162/412 (39.32)*	486/996 (48.80)*	2,091/3,696 (56.57)	200/373 (53.62)	220/399 (55.14)	<0.001
Live birth rate #	200/560 (35.71)*	159/412 (38.59)*	462/995 (46.43)*	2,017/3,691 (54.65)	196/373 (52.55)	214/398 (53.77)	<0.001
Early miscarriage rate	58/265 (21.89)*	50/212 (23.58)*	115/601 (19.13)	382/2,473 (15.45)	30/230 (13.04)	35/255 (13.73)	<0.001

Data are presented as n/N (%). *P* values were calculated using chi-square.

*P < 0.01 compared with the 8-cell group, corresponding to Bonferroni correction for five comparisons (0.05/5).

#Eight cycles were lost to follow-up for live birth outcomes.

**Figure 2:**
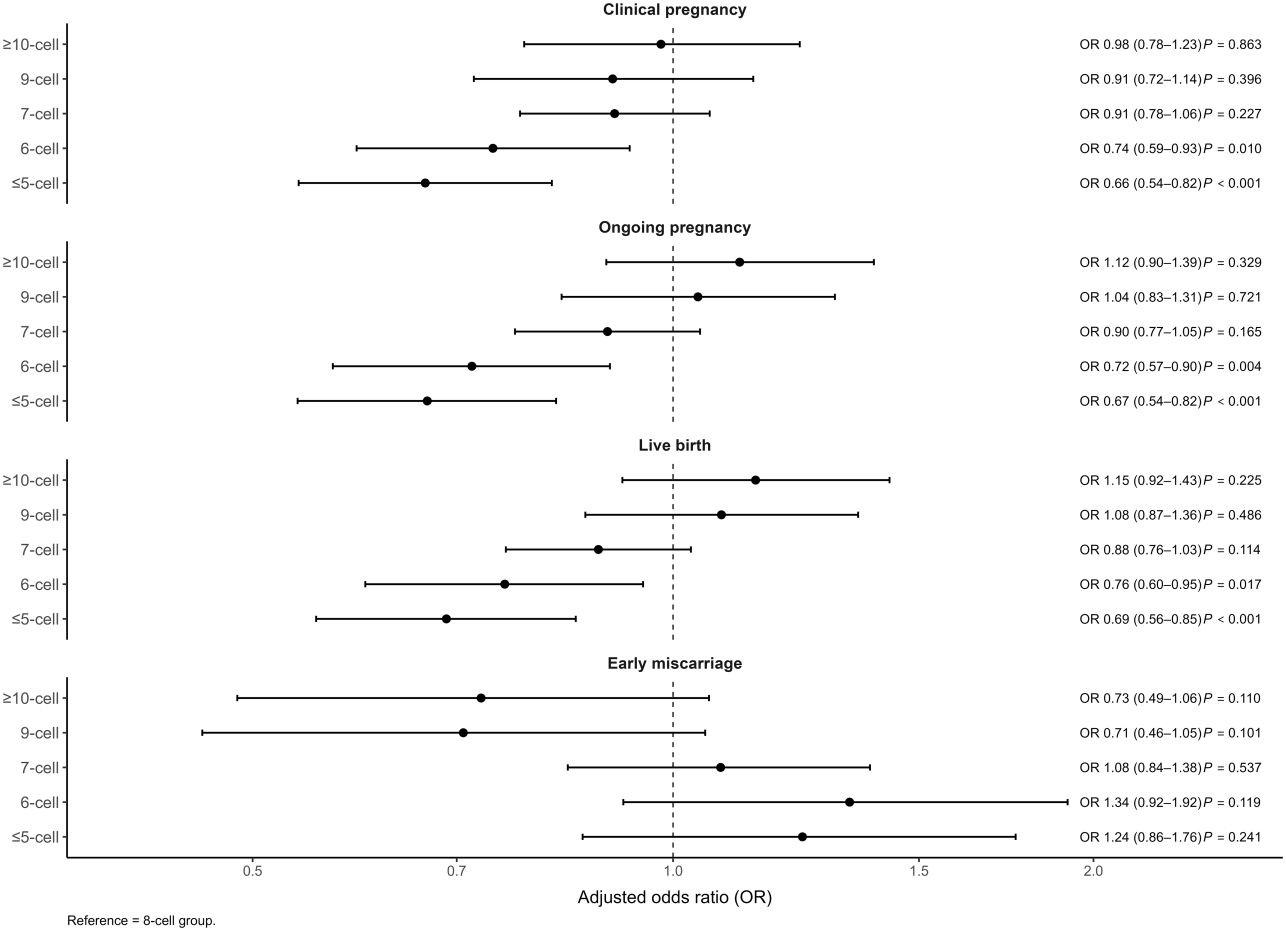
Adjusted odds ratios for pregnancy outcomes according to Day-3 embryo cell number.

In multivariable logistic regression analyses adjusted for relevant covariates, blastocysts derived from Day-3 embryos with ≤5 or 6 cells remained significantly associated with poorer pregnancy outcomes than those derived from 8-cell embryos (**Figure 2**). Specifically, the odds of live birth were reduced in both the ≤5-cell group (aOR, 0.69; 95% CI, 0.56–0.85) and the 6-cell group (aOR, 0.76; 95% CI, 0.60–0.95). Similar associations were observed for clinical pregnancy (≤5-cell group: aOR, 0.66; 95% CI, 0.54–0.82; 6-cell: aOR, 0.74; 95% CI, 0.59–0.93) and ongoing pregnancy (≤5-cell group: aOR, 0.67; 95% CI, 0.54–0.82; 6-cell: aOR, 0.72; 95% CI, 0.57–0.90). However, the associations for the 7-cell group were no longer statistically significant after adjustment. Early miscarriage rates were also comparable across Day-3 cell number groups after adjustment for relevant covariates.

Sensitivity analyses were performed by stratifying the cohort according to blastocyst quality and blastocyst day (**Table 3**). Among high-quality blastocysts, blastocysts derived from ≤5-cell embryos remained associated with lower odds of clinical pregnancy, ongoing pregnancy, and live birth than those derived from 8-cell embryos. The 6-cell group was also associated with reduced odds of ongoing pregnancy, although the associations with clinical pregnancy and live birth were attenuated. By contrast, ≥10-cell embryos were associated with higher odds of live birth among high-quality blastocysts, whereas no comparable increases were observed for clinical pregnancy or ongoing pregnancy. Similar patterns were observed among low-quality blastocysts, in which both ≤5-cell and 6-cell groups were consistently associated with lower odds of clinical pregnancy, ongoing pregnancy, and live birth than 8-cell group. The ≥10-cell group also exhibited lower odds of clinical pregnancy, ongoing pregnancy, and live birth, although the number of cycles in this category was relatively small. When the analysis was restricted to Day-5 blastocysts, the adverse associations for ≤5-cell and 6-cell groups persisted. Compared with the 8-cell group, the ≤5-cell group exhibited lower odds of clinical pregnancy, ongoing pregnancy, and live birth, and similar reductions were observed in the 6-cell group. When the analysis was restricted to Day-6 blastocysts, the ≤5-cell group remained associated with lower odds of ongoing pregnancy and live birth, whereas the 6-cell group showed a tendency toward lower odds of clinical pregnancy, ongoing pregnancy, and live birth, although none of the associations reached statistical significance.

**Table 3:** Stratified analyses of the associations between Day-3 cell number and pregnancy outcomes.

Variable	Measure	≤5-cell	6-cell	7-cell	8-cell	9-cell	≥10-cell	P value
High-quality blastocysts								
Clinical pregnancy	Rate	178/300 (59.33)*	146/243 (60.08)*	492/767 (64.15)	2348/3447 (68.12)	200/304 (65.79)	230/330 (69.70)	0.002
	aOR	0.72 (0.56–0.95)#	0.76 (0.57–1.00)	0.88 (0.74–1.04)	Reference	0.92 (0.72–1.19)	1.10 (0.85–1.42)	
Ongoing pregnancy	Rate	147/300 (49.00)*	115/243 (47.33)*	408/767 (53.19)	1991/3447 (57.76)	178/304 (58.55)	203/330 (61.52)	<0.001
	aOR	0.73 (0.56–0.95) #	0.73 (0.55–0.96) #	0.89 (0.75–1.05)	Reference	1.08 (0.85–1.39)	1.26 (0.99–1.60)	
Live birth	Rate	141/299 (47.16)*	113/243 (46.50)*	389/766 (50.78)	1922/3442 (55.84)	174/304 (57.24)	197/329 (59.88)	<0.001
	aOR	0.75 (0.58–0.97)#	0.77 (0.59–1.02)	0.88 (0.75–1.04)	Reference	1.12 (0.88–1.44)	1.28 (1.01–1.64)#	
Early miscarriage	Rate	31/178 (17.42)	31/146 (21.23)	84/492 (17.07)	357/2348 (15.20)	22/200 (11.00)	27/230 (11.74)	0.054
	aOR	1.14 (0.72–1.77)	1.33 (0.84–2.04)	1.05 (0.80–1.38)	Reference	0.64 (0.39–1.00)	0.63 (0.40–0.96) #	
Low-quality blastocysts								
Clinical pregnancy	Rate	87/261 (33.33)*	66/169 (39.05)	109/229 (47.60)	125/249 (50.20)	30/69 (43.48)	25/69 (36.23)	0.002
	aOR	0.51 (0.34–0.75)#	0.60 (0.39–0.92)#	0.90 (0.61–1.32)	Reference	0.80 (0.46–1.41)	0.53 (0.29–0.93)#	
Ongoing pregnancy	Rate	60/261 (22.99)*	47/169 (27.81)*	78/229 (34.06)	100/249 (40.16)	22/69 (31.88)	17/69 (24.64)	<0.001
	aOR	0.44 (0.29–0.68)#	0.54 (0.34–0.85)#	0.77 (0.51–1.15)	Reference	0.76 (0.41–1.37)	0.48 (0.25–0.89)#	
Live birth	Rate	59/261 (22.61)*	46/169 (27.22)	73/229 (31.88)	95/249 (38.15)	22/69 (31.88)	17/69 (24.64)	0.005
	aOR	0.47 (0.31–0.72)#	0.57 (0.36–0.90)#	0.74 (0.49–1.12)	Reference	0.80 (0.44–1.44)	0.51 (0.26–0.94)#	
Early miscarriage	Rate	27/87 (31.03)	19/66 (28.79)	31/109 (28.44)	25/125 (20.00)	8/30 (26.67)	8/25 (32.00)	0.496
	aOR	1.88 (0.93–3.81)	1.60 (0.75–3.38)	1.45 (0.74–2.85)	Reference	1.28 (0.46–3.36)	1.57 (0.54–4.33)	
Day-5 blastocysts								
Clinical pregnancy	Rate	101/205 (49.27)*	128/229 (55.90)*	465/739 (62.92)*	2226/3275 (67.97)	204/312 (65.38)	236/357 (66.11)	<0.001
	aOR	0.53 (0.39–0.72)#	0.69 (0.51–0.92)#	0.87 (0.73–1.04)	Reference	0.95 (0.74–1.23)	0.99 (0.78–1.26)	
Ongoing pregnancy	Rate	83/205 (40.49)*	95/229 (41.48)*	384/739 (51.96)*	1877/3275 (57.31)	178/312 (57.05)	208/357 (58.26)	<0.001
	aOR	0.60 (0.43–0.82)#	0.63 (0.47–0.84)#	0.90 (0.76–1.07)	Reference	1.09 (0.85–1.40)	1.19 (0.95–1.51)	
Live birth	Rate	81/205 (39.51)*	93/229 (40.61)*	367/738 (49.73)*	1810/3270 (55.35)	175/312 (56.09)	203/356 (57.02)	<0.001
	aOR	0.63 (0.46–0.86)#	0.66 (0.50–0.89)#	0.89 (0.75–1.06)	Reference	1.15 (0.90–1.47)	1.24 (0.98–1.57)	
Early miscarriage	Rate	18/101 (17.82)	33/128 (25.78)*	81/465 (17.42)	349/2226 (15.68)	26/204 (12.75)	28/236 (11.86)	0.011
	aOR	1.01 (0.56–1.74)	1.54 (0.97–2.39)	1.01 (0.75–1.33)	Reference	0.70 (0.44–1.08)	0.61 (0.39–0.92)#	
Day-6 blastocysts								
Clinical pregnancy	Rate	164/356 (46.07)*	84/183 (45.90)*	136/257 (52.92)	247/421 (58.67)	26/61 (42.62)	19/42 (45.24)	0.003
	aOR	0.78 (0.57–1.07)	0.82 (0.56–1.20)	0.99 (0.70–1.40)	Reference	0.67 (0.37–1.19)	0.85 (0.43–1.65)	
Ongoing pregnancy	Rate	124/356 (34.83)*	67/183 (36.61)*	102/257 (39.69)*	214/421 (50.83)	22/61 (36.07)	12/42 (28.57)*	<0.001
	aOR	0.66 (0.48–0.90)#	0.80 (0.54–1.17)	0.80 (0.56–1.13)	Reference	0.73 (0.40–1.31)	0.57 (0.27–1.16)	
Live birth	Rate	119/355 (33.52)*	66/183 (36.07)*	95/257 (36.96)*	207/421 (49.17)	21/61 (34.43)	11/42 (26.19)*	<0.001
	aOR	0.67 (0.48–0.92)#	0.82 (0.55–1.21)	0.76 (0.53–1.07)	Reference	0.71 (0.39–1.28)	0.53 (0.24–1.08)	
Early miscarriage	Rate	40/164 (24.39)*	17/84 (20.24)	34/136 (25.00)*	33/247 (13.36)	4/26 (15.38)	7/19 (36.84)*	0.012
	aOR	1.86 (1.07–3.25)#	1.39 (0.68–2.76)	1.76 (0.98–3.16)	Reference	0.86 (0.23–2.56)	2.55 (0.84–7.28)	

Data are presented as n/N (%), or adjusted odds ratio (aOR) with 95% confidence interval.

The *P* value was calculated using the Pearson chi-square test for the overall comparison among Day-3 cell-number groups.

*P < 0.01 compared with the 8-cell group using the Pearson chi-square test, corresponding to Bonferroni correction for five comparisons (0.05/5).

#*P* < 0.05 compared with the 8-cell group in the multivariable logistic regression model.

As an additional sensitivity analysis, propensity score matching (PSM) was performed for each Day-3 cell number group, using the 8-cell group as the reference. After matching, covariate balance was substantially improved across all comparisons, as indicated by the SMDs (**Supplementary Figure 1**). In the matched analyses, blastocysts derived from ≤5-cell and 6-cell embryos remained associated with lower odds of clinical pregnancy, ongoing pregnancy, and live birth than those derived from 8-cell embryos (**Supplementary Figure 2**).

### 3.3 Obstetric and perinatal outcomes across Day-3 cell number groups

Obstetric and perinatal outcomes were evaluated in 3191 singleton live-birth cycles (**Table 4**). No significant differences were observed across the Day-3 cell number groups in gestational age at delivery, cesarean delivery, gestational diabetes mellitus (GDM), gestational hypertension (GH), postpartum hemorrhage (PPH), neonatal sex, birth weight, male birth weight, female birth weight, birth weight <1000 g, birth weight <1500 g, birth weight <2500 g, neonatal incubator admission, or birth defects. In the adjusted analysis, the overall association between Day-3 cell number and preterm birth reached statistical significance (P = 0.039); however, this finding was driven primarily by lower odds in the 7-cell group than in the 8-cell group (aOR, 0.56; 95% CI, 0.35–0.87), whereas no significant differences were observed for the other groups (**Table 5**). Importantly, neither the ≤5-cell nor the 6-cell group showed increased odds of preterm birth or any other evaluated adverse obstetric or perinatal outcomes compared with the 8-cell group.

**Table 4:** Obstetric and perinatal outcomes among singleton live births.

Variable	≤5-cell (N = 199)	6-cell (N = 156)	7-cell (N = 456)	8-cell (N = 1979)	9-cell (N = 193)	≥10-cell (N = 208)	*P* value
Gestational days	275.00 (269.00–280.00)	274.00 (269.00–280.00)	274.00 (269.00–279.00)	274.00 (269.00–280.00)	274.00 (268.00–279.00)	274.00 (269.00–279.00)	0.696
Preterm birth	13/199 (6.53)	8/156 (5.13)	23/456 (5.04)	168/1,979 (8.49)	21/193 (10.88)	16/208 (7.69)	0.058
Extremely preterm birth	1/199 (0.50)	1/156 (0.64)	0/456 (0.00)	7/1,979 (0.35)	1/193 (0.52)	0/208 (0.00)	0.404
Cesarean delivery	126/199 (63.32)	99/156 (63.46)	289/456 (63.38)	1,247/1,979 (63.01)	109/193 (56.48)	135/208 (64.90)	0.571
Complications							
GDM	36/199 (18.09)	26/156 (16.67)	73/456 (16.01)	308/1,979 (15.56)	31/193 (16.06)	42/208 (20.19)	0.608
GH	15/199 (7.54)	9/156 (5.77)	34/456 (7.46)	109/1,979 (5.51)	6/193 (3.11)	11/208 (5.29)	0.271
PPH	4/199 (2.01)	4/156 (2.56)	9/456 (1.97)	31/1,979 (1.57)	1/193 (0.52)	4/208 (1.92)	0.619
Sex							0.198
Male	110/199 (55.28)	84/156 (53.85)	278/456 (60.96)	1,105/1,979 (55.84)	117/193 (60.62)	127/208 (61.06)	
Female	89/199 (44.72)	72/156 (46.15)	178/456 (39.04)	874/1,979 (44.16)	76/193 (39.38)	81/208 (38.94)	
Birth weight (g)#	3300 (3000–3500)	3200 (2980–3480)	3250 (3000–3500)	3207.50 (2950–3500)	3300 (3000–3550)	3250 (3000–3500)	0.500
Male birth weight (g)	3300 (3030–3500)	3200 (3000–3450)	3300 (3050–3550)	3270 (3000–3550)	3300 (3000–3630)	3250 (3000–3500)	0.536
Female birth weight (g)	3250 (2900–3550)	3200 (2940–3500)	3150 (2870–3490)	3150 (2900–3430)	3250 (2950–3475)	3250 (2950–3500)	0.298
Birth weight <1000	1/199 (0.50)	1/156 (0.64)	1/456 (0.22)	7/1,978 (0.35)	2/193 (1.04)	0/208 (0.00)	0.394
Birth weight <1500	2/199 (1.01)	3/156 (1.92)	4/456 (0.88)	19/1,978 (0.96)	3/193 (1.55)	1/208 (0.48)	0.678
Birth weight <2500	6/199 (3.02)	6/156 (3.85)	21/456 (4.61)	115/1,978 (5.81)	13/193 (6.74)	11/208 (5.29)	0.432
Neonatal incubator admission	7/199 (3.52)	2/156 (1.28)	14/456 (3.07)	89/1,979 (4.50)	13/193 (6.74)	7/208 (3.37)	0.111
Birth defect	0/199 (0.00)	1/156 (0.64)	1/456 (0.22)	8/1,979 (0.40)	0/193 (0.00)	1/208 (0.48)	0.788

Continuous variables are presented as median (IQR), and categorical variables are presented as n/N (%).

Overall *P* values were calculated using the Kruskal–Wallis test for continuous variables and Pearson’s chi-square test or Fisher’s exact test for categorical variables.

Pairwise comparisons with the 8-cell group were performed using a Bonferroni-corrected significance threshold of P < 0.01 (0.05/5).

GDM, gestational diabetes mellitus; GH, gestational hypertension; PPH, postpartum hemorrhage.

#Birth weight was missing in one cycle.

**Table 5:** Adjusted associations between Day-3 cell-number groups and obstetric and perinatal outcomes among singleton live births.

Variable	Effect estimate	≤5-cell	6-cell	7-cell	8-cell	9-cell	≥10-cell	P value
Gestational days	Adjusted β (95% CI)	1.42 (-0.41–3.26)	0.49 (-1.54–2.53)	1.32 (0.05–2.59)*	Reference	-1.25 (-3.08–0.59)	0.47 (-1.30–2.24)	0.122
Preterm birth	Adjusted OR (95% CI)	0.72 (0.38–1.27)	0.58 (0.25–1.14)	0.56 (0.35–0.87)*	Reference	1.35 (0.81–2.14)	0.92 (0.52–1.53)	0.039
Cesarean delivery	Adjusted OR (95% CI)	0.90 (0.66–1.24)	0.94 (0.66–1.34)	0.99 (0.80–1.24)	Reference	0.76 (0.56–1.04)	1.06 (0.78–1.45)	0.597
GDM	Adjusted OR (95% CI)	1.21 (0.81–1.77)	1.10 (0.69–1.69)	1.03 (0.77–1.36)	Reference	1.05 (0.69–1.56)	1.39 (0.95–1.98)	0.603
GH	Adjusted OR (95% CI)	1.31 (0.70–2.30)	1.08 (0.49–2.10)	1.43 (0.94–2.14)	Reference	0.53 (0.20–1.15)	1.00 (0.49–1.83)	0.257
PPH	Adjusted OR (95% CI)	1.11 (0.32–2.96)	1.49 (0.43–3.94)	1.17 (0.52–2.41)	Reference	0.31 (0.02–1.49)	1.29 (0.38–3.35)	0.713
Birth weight (g)	Adjusted β (95% CI)	51.00 (-22.47–124.47)	-0.69 (-82.32–80.93)	37.73 (-13.12–88.59)	Reference	7.59 (-65.79–80.98)	42.94 (-28.12–113.99)	0.488
Male birth weight (g)	Adjusted β (95% CI)	1.26 (-98.66–101.19)	-46.44 (-159.52–66.64)	38.29 (-28.05–104.63)	Reference	10.44 (-85.30–106.18)	-7.00 (-99.32–85.33)	0.806
Female birth weight (g)	Adjusted β (95% CI)	104.37 (-3.06–211.80)	56.12 (-60.95–173.19)	19.38 (-59.68–98.45)	Reference	-9.82 (-123.79–104.15)	104.41 (-6.23–215.05)	0.211
Birth weight <1500 g	Adjusted OR (95% CI)	1.14 (0.18–4.14)	2.20 (0.50–6.84)	0.93 (0.26–2.56)	Reference	1.77 (0.41–5.35)	0.51 (0.03–2.50)	0.747
Birth weight <2500 g	Adjusted OR (95% CI)	0.47 (0.18–1.01)	0.64 (0.25–1.38)	0.77 (0.46–1.23)	Reference	1.22 (0.64–2.13)	0.93 (0.46–1.69)	0.312
Neonatal incubator admission	Adjusted OR (95% CI)	0.76 (0.31–1.58)	0.27 (0.04–0.87)	0.66 (0.36–1.14)	Reference	1.55 (0.81–2.74)	0.78 (0.32–1.60)	0.082

Values are adjusted odds ratios (OR) with 95% confidence intervals (CI) for binary outcomes and adjusted regression coefficients (β) with 95% CI for continuous outcomes. The 8-cell group was used as the reference group.

Overall *P* values represent the association between Day-3 cell-number group and each outcome in the multivariable regression model.

*P < 0.05 compared with the 8-cell group.

GDM, gestational diabetes mellitus; GH, gestational hypertension; PPH, postpartum hemorrhage.

## 4 Discussion

The present study focused on two clinically relevant questions: first, whether deviation from the optimal 8-cell stage on Day 3 compromises pregnancy outcomes despite subsequent blastocyst formation; and second, whether blastocysts derived from embryos that exhibited suboptimal development on Day 3 increase risks of adverse obstetric and perinatal outcomes. In this large cohort of 6437 frozen–thawed SBT cycles, blastocysts derived from Day-3 embryos with ≤5 or 6 cells were associated with significantly lower odds of clinical pregnancy, ongoing pregnancy, and live birth than those derived from 8-cell embryos, and these associations persisted after multivariable adjustment and following PSM. In contrast, among 3191 singleton live births, neither the ≤5-cell nor the 6-cell group showed significant differences in gestational age at delivery, cesarean delivery, GDM, GH, PPH, neonatal sex, birth weight, male birth weight, female birth weight, birth weight <1000 g, birth weight <1500 g, birth weight <2500 g, neonatal incubator admission, or birth defects. Taken together, these findings indicate that Day-3 cell number retains clinical predictive value for the probability of achieving pregnancy and live birth even after the embryo has reached the blastocyst stage, whereas its influence does not appear to extend to short-term obstetric and perinatal outcomes among singleton live-birth cycles.

Optimal cleavage-stage development on Day 3 is generally considered to correspond to 7–9 cells, with 8-cell embryos exhibiting the highest implantation potential and chromosomal euploidy rates (1, 17). In agreement with previous results, the 8-cell group demonstrated the highest live birth rate in the present study, supporting its use as the reference group. The inferior outcomes observed with the ≤ 5-cell and 6-cell groups are consistent with several previous studies reporting reduced implantation and live birth rates for slow-cleaving embryos, although the exact cell number threshold varied across studies (10, 13, 18). Specifically, Wu et al. reported significantly lower live birth rates for ≤ 5-cell embryos (13). In comparison, Zhao et al. (10) and Zhou et al. (18) observed similar reductions for embryos with ≤6 and <7 cells, respectively. The present study extended these findings in a larger SBT cohort by categorizing Day-3 cell number into six groups (≤5, 6, 7, 8, 9, and ≥10 cells) for a more detailed assessment and by examining the associations using multivariable regression, stratified sensitivity analyses, and PSM. Overall, our findings suggest that slow cleavage on Day 3, particularly to ≤5 cells, may indicate reduced intrinsic developmental potential, even when the embryo subsequently reaches the blastocyst stage.

The findings for the 6-cell group deserve particular consideration. Although blastocysts derived from 6-cell embryos were associated with poorer pregnancy outcomes in the overall and PSM analyses, the strength and consistency of this association varied across subgroup analyses. Among low-quality blastocysts and Day-5 blastocysts, the 6-cell group showed significantly lower odds of clinical pregnancy, ongoing pregnancy, and live birth; among high-quality blastocysts, only ongoing pregnancy reached statistical significance. Among Day-6 blastocysts, point estimates remained directionally consistent with reduced odds across all three outcomes, although none reached statistical significance. Collectively, these findings indicate that the impact of Day-3 cell number on pregnancy outcomes may be modified by subsequent blastocyst quality and blastocyst day.

Embryos with ≤5 cells and those with 6 cells on Day 3 may represent different degrees of developmental delay (19), and their effects on outcomes after SBT may therefore differ. Compared with 6-cell embryos, ≤5-cell embryos may reflect more severe developmental compromise that is not fully overcome by biological selection during extended culture. By contrast, the attenuation of the association among high-quality blastocysts in the 6-cell group may reflect a selection effect during blastocyst culture. A 6-cell embryo that subsequently forms a high-quality blastocyst may represent a selected “top performer” with preserved developmental competence, thereby mitigating the adverse impact associated with a lower Day-3 cell number (11, 12, 20). Conversely, among Day-6 blastocysts, an 8-cell embryo in the reference group that subsequently developed into a Day-6 blastocyst may represent a selected “marginal performer”. This may partly explain why the 6-cell group showed a similar adverse direction of effect compared with the 8-cell group in the Day-6 blastocyst subgroup, but without statistically significant differences. Reduced statistical power after stratification may also have contributed to this result. When Day-3 cell number increased to 7 cells, the difference in pregnancy outcomes compared with the 8-cell group was no longer significant after adjustment for relevant covariates. Collectively, these findings suggest that 6-cell embryos occupy an intermediate or transitional position within the slow-cleavage spectrum, with developmental implications that fall between those of ≤5-cell and 7-cell embryos. Such a graded pattern may have been obscured in previous studies in which embryos with ≤6 or ≤7 cells were grouped into a single slow-cleavage category.

Several studies have reported that rapidly cleaving embryos with higher than optimal cell numbers are associated with higher live birth rates (19, 21), contrasting with the traditional view that 8-cell embryos are most favorable (1, 3). However, we did not observe this benefit in our overall analysis or after PSM. This finding is consistent with those of Zhao et al. and Zhou et al. (10, 18), and may be interpreted by the recent evidence suggesting that rapidly cleaving embryos that achieve blastulation present similar euploidy rates and clinical outcomes to normally cleaving embryos (4). Nevertheless, in the present study, ≥10-cell embryos were associated with higher odds of live birth among high-quality blastocysts, whereas the same group showed lower odds of clinical pregnancy, ongoing pregnancy, and live birth among low-quality blastocysts. This heterogeneity suggests that accelerated cleavage is not uniformly adverse and may carry dual implications. When accompanied by good blastocyst morphology, it may reflect robust developmental kinetics and enhanced implantation potential (22). When accompanied by poor blastocyst morphology, however, it may instead reflect abnormal division patterns and cell-cycle dysregulation, manifesting as poorer pregnancy outcomes (23).

Another important contribution of the present study is the extension of the evaluation from pregnancy outcomes to obstetric and perinatal outcomes. Previous investigations of Day-3 cell number have focused predominantly on implantation, clinical pregnancy, miscarriage, and live birth, with limited attention to obstetric and perinatal outcomes. In the present cohort of 3191 singleton live births, neither the ≤5-cell nor the 6-cell group showed significant increases in preterm birth, cesarean delivery, GDM, GH, PPH, low birth weight, neonatal incubator admission, or birth defects compared with the 8-cell group. This finding is consistent with a recent study which also reported no significant effect of Day-3 embryo cell number on neonatal outcomes in Day-5 single SBT cycles (24). Indeed, a growing body of evidence indicates that embryo morphology is more strongly associated with implantation and live birth than with downstream obstetric or perinatal outcomes. For example, Oron et al. reported that poor embryo quality was associated with lower pregnancy and live birth rates but was not independently associated with adverse obstetric or perinatal outcomes among singleton births after single embryo transfer (25). Similarly, Bakkensen et al. found that blastocyst expansion and trophectoderm quality were associated with clinical pregnancy and live birth, whereas few associations with perinatal outcomes were identified (26). A large multicenter cohort study also demonstrated that blastocyst developmental stage and morphology were not associated with preterm birth or birth weight among singleton live births (27). However, the lower odds of preterm birth observed in the 7-cell group compared with the 8-cell group in present study should be interpreted cautiously, as this association did not follow a biologically plausible pattern across Day-3 cell number groups. It may represent a chance finding arising from differences in sample size, multiple testing, or unmeasured confounding.

This study has several methodological strengths. The large sample size of 6437 frozen–thawed SBT cycles and 3191 singleton live births provided adequate statistical power for both pregnancy and perinatal analyses. The range of outcomes evaluated was comprehensive, encompassing clinical pregnancy, ongoing pregnancy, live birth, early miscarriage, and a broad panel of obstetric and perinatal outcomes. The granular classification of Day-3 cell number into six groups enabled the identification of subgroup-specific patterns that would be obscured by broader categorization schemes. The application of multivariable regression, stratified sensitivity analyses, and PSM strengthened the credibility of the observed associations. Furthermore, the restriction to frozen–thawed SBT cycles minimized confounding by the complications associated with multiple embryo transfer. However, several limitations should be acknowledged. First, because only embryos that developed into available blastocysts and then were selected for SBT were included, the findings should be interpreted as conditional associations rather than as the total effect of Day-3 cell number on clinical outcomes. Blastocyst day and quality may partly lie on the developmental pathway between Day-3 cell number and pregnancy outcomes; therefore, adjustment for these variables estimates associations conditional on subsequent blastocyst development and morphology. This model better addresses the clinical question of whether Day-3 cell number remains associated with pregnancy outcomes among transferred blastocysts with comparable morphology, but these estimates should not be interpreted as the total effect of Day-3 cell number across all cleavage-stage embryos. Second, the single-center retrospective design may be susceptible to selection bias and residual confounding, despite multivariable adjustment, stratified analyses, and propensity score matching. Finally, some subgroup analyses, particularly for the ≤5-cell and ≥10-cell groups, had limited sample sizes, resulting in wider confidence intervals and reduced precision. Further studies are needed to confirm these findings.

The present findings support a balanced approach to embryo selection. Blastocysts derived from Day-3 embryos with ≤5 or 6 cells should not be discarded, as a meaningful proportion can still result in successful pregnancy and live birth. However, when multiple blastocysts of comparable morphological quality and developmental timing are available, those originating from 8-cell embryos may be reasonably prioritized. For blastocysts derived from 6 cells, the decision should integrate blastocyst quality and developmental day, rather than relying solely on Day-3 cell number as a criterion for exclusion. In patient counselling, clinicians can communicate that blastocysts from slow-cleaving embryos carry a lower probability of pregnancy success, but that the present study does not indicate increased obstetric or perinatal risk among singleton live births. This balanced message avoids overly negative attitudes of slow-cleaving blastocysts while remaining consistent with the available evidence.

In summary, blastocysts derived from Day-3 embryos with ≤5 or 6 cells were associated with reduced clinical pregnancy, ongoing pregnancy, and live birth rates after frozen-thawed SBT compared with those derived from 8-cell embryos. However, this association did not appear to extend to the short-term obstetric or perinatal safety of live-born singletons. These findings may help inform blastocyst selection strategies and support evidence-based patient counselling in clinical practice.

## Data Availability

The raw data supporting the conclusions of this article will be made available by the authors, without undue reservation.

## Supplementary material

Supplementary Figure 1 Covariate balance before and after propensity score matching. Love plots showing the absolute standardized mean differences (SMDs) for baseline and cycle characteristics before and after propensity score matching (PSM). The vertical dashed line indicates an SMD of 0.10, which was used as the threshold for acceptable covariate balance. OPU, oocyte pick-up; BMI, body mass index; AMH, anti-Müllerian hormone; ICSI, intracytoplasmic sperm injection; IVF, in vitro fertilization; HRT, hormone replacement therapy; NC, natural cycle; OI, ovulation induction.

Supplementary Figure 2 Propensity score matched analysis of pregnancy outcomes according to Day-3 cell number. Values on the right indicate the matched sample size in each comparison (N = treated/reference), odds ratios (ORs) with 95% confidence intervals (CIs) and corresponding P value.
