# Supplementary figures and images for: Impact of Day-3 embryo cell number on pregnancy, obstetric and perinatal outcomes in frozen-thawed single blastocyst transfer cycles
Source: Front Endocrinol (Lausanne). 2026 Jun 22;17:1777070. doi: 10.3389/fendo.2026.1777070 (PMC13333433; doi:10.3389/fendo.2026.1777070)

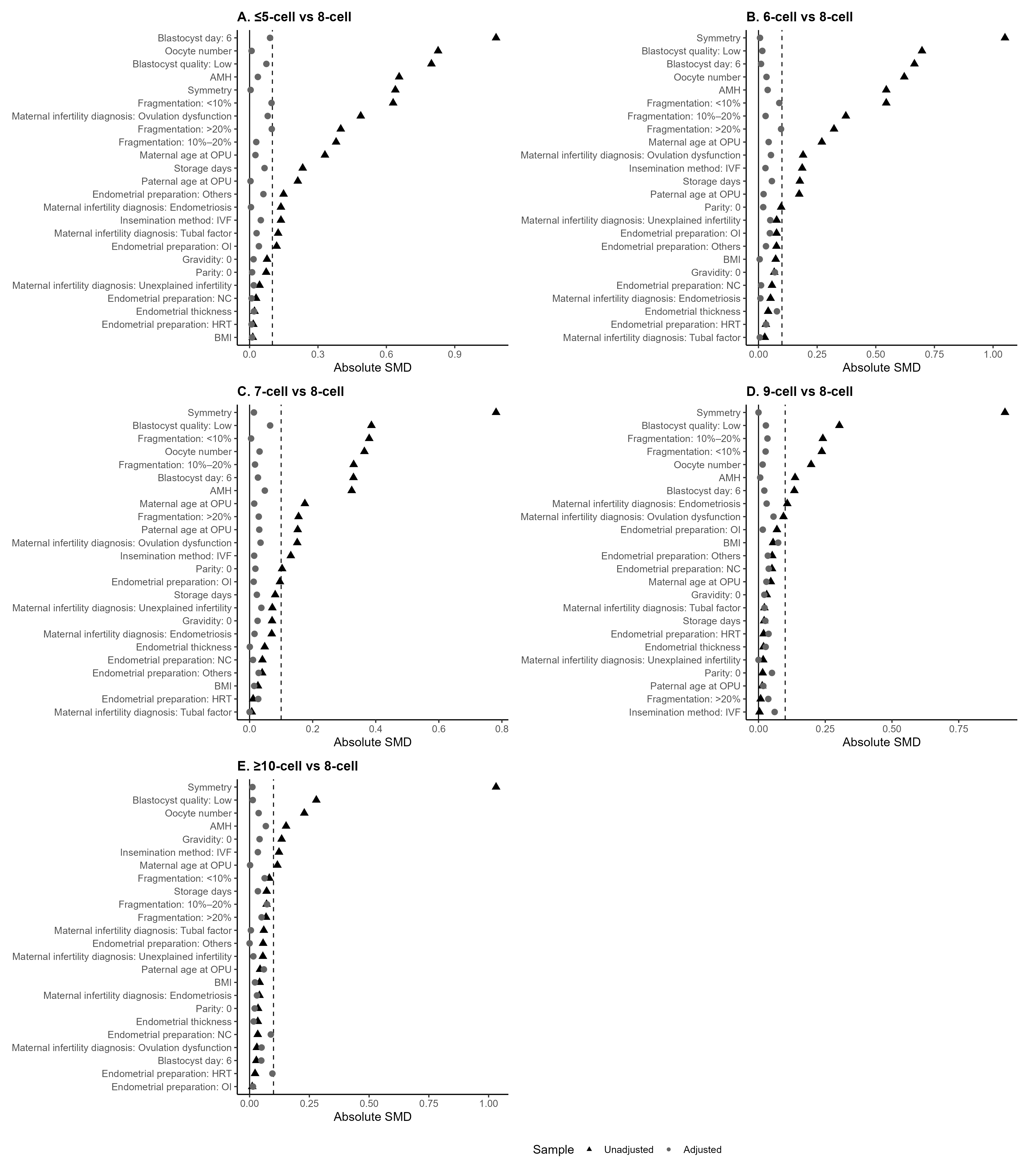

Supplement: Supplementary Figure 1 — Covariate balance before and after propensity score matching. Love plots showing the absolute standardized mean differences (SMDs) for baseline and cycle characteristics before and after propensity score matching (PSM). The vertical dashed line indicates an SMD of 0.10, which was used as the threshold for acceptable covariate balance. OPU, oocyte pick-up; BMI, body mass index; AMH, anti-Müllerian hormone; ICSI, intracytoplasmic sperm injection; IVF, in vitro fertilization; HRT, hormone replacement therapy; NC, natural cycle; OI, ovulation induction. [file Image1.tiff]

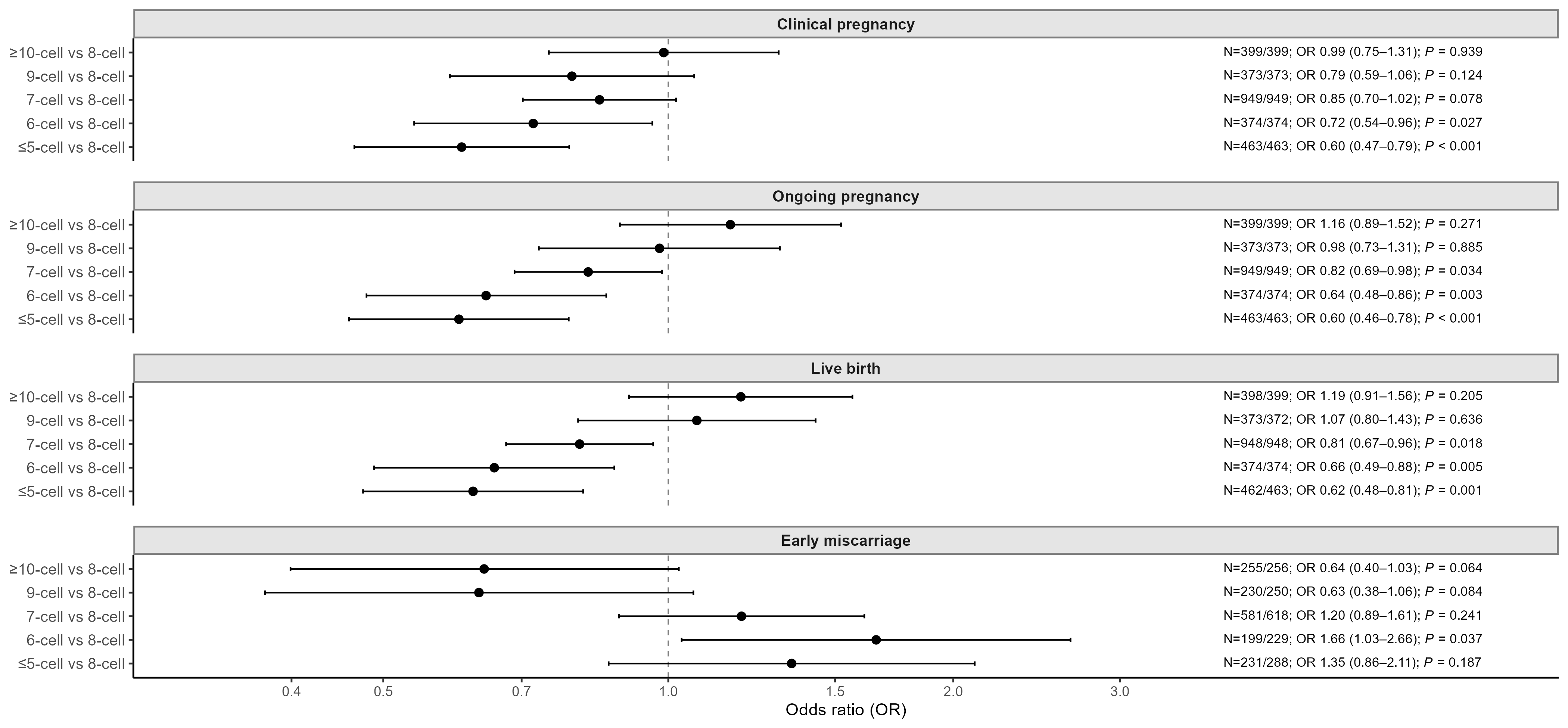

Supplement: Supplementary Figure 2 — Propensity score matched analysis of pregnancy outcomes according to Day-3 cell number. Values on the right indicate the matched sample size in each comparison (N = treated/reference), odds ratios (ORs) with 95% confidence intervals (CIs) and corresponding P value. [file Image2.tiff]
