# Supplementary material for: Impact of Day-3 embryo cell number on pregnancy, obstetric and perinatal outcomes in frozen-thawed single blastocyst transfer cycles
Source: Front Endocrinol (Lausanne). 2026 Jun 22;17:1777070. doi: 10.3389/fendo.2026.1777070 (PMC13333433; doi:10.3389/fendo.2026.1777070)
Supplement: Supplementary file 3 [file Table1.docx]

**Supplementary Table S1.** Standardized mean differences (SMDs) of baseline characteristics compared with the 8-cell group.

| **Variable** | **≤5-cell vs 8-cell** | **6-cell vs 8-cell** | **7-cell vs 8-cell** | **9-cell vs 8-cell** | **≥10-cell vs 8-cell** |
| --- | --- | --- | --- | --- | --- |
| Maternal age at ET (years) | 0.354 | 0.288 | 0.188 | 0.050 | 0.115 |
| Paternal age at ET (years) | 0.229 | 0.192 | 0.163 | 0.014 | 0.047 |
| Maternal age at OPU (years) | 0.340 | 0.279 | 0.183 | 0.048 | 0.121 |
| Paternal age at OPU (years) | 0.223 | 0.184 | 0.158 | 0.014 | 0.044 |
| Infertility duration (years) | 0.120 | 0.090 | 0.065 | 0.009 | 0.047 |
| BMI (kg/m2) | 0.014 | 0.076 | 0.026 | 0.055 | 0.042 |
| AMH (ng/mL) | 0.568 | 0.463 | 0.305 | 0.125 | 0.152 |
| Gravidity |  |  |  |  |  |
| 0 | 0.077 | 0.067 | 0.072 | 0.031 | 0.132 |
| ≥1 | 0.077 | 0.067 | 0.072 | 0.031 | 0.132 |
| Parity |  |  |  |  |  |
| 0 | 0.075 | 0.101 | 0.108 | 0.016 | 0.035 |
| ≥1 | 0.075 | 0.101 | 0.108 | 0.016 | 0.035 |
| Maternal infertility diagnosis |  |  |  |  |  |
| Unexplained infertility | 0.044 | 0.078 | 0.073 | 0.018 | 0.056 |
| Ovulation dysfunction | 0.381 | 0.175 | 0.142 | 0.090 | 0.030 |
| Tubal factor | 0.127 | 0.027 | 0.007 | 0.022 | 0.059 |
| Endometriosis | 0.148 | 0.053 | 0.073 | 0.114 | 0.042 |
| Oocyte number | 0.785 | 0.587 | 0.363 | 0.189 | 0.235 |
| Insemination method |  |  |  |  |  |
| ICSI | 0.144 | 0.197 | 0.136 | 0.004 | 0.117 |
| IVF | 0.144 | 0.197 | 0.136 | 0.004 | 0.117 |
| Day-3 fragmentation |  |  |  |  |  |
| <10% | 0.000 | 0.000 | 0.000 | 0.000 | 0.000 |
| 10%-20% | 0.443 | 0.434 | 0.381 | 0.271 | 0.068 |
| >20% | 0.000 | 0.000 | 0.000 | 0.000 | 0.000 |
| Day-3 symmetry |  |  |  |  |  |
| Uneven | 0.646 | 0.989 | 0.774 | 0.892 | 0.975 |
| Even | 0.646 | 0.989 | 0.774 | 0.892 | 0.975 |
| Blastocyst day |  |  |  |  |  |
| 5 | 1.276 | 0.792 | 0.377 | 0.144 | 0.028 |
| 6 | 1.276 | 0.792 | 0.377 | 0.144 | 0.028 |
| Storage days | 0.287 | 0.198 | 0.094 | 0.023 | 0.075 |
| Endometrial thickness (mm) | 0.022 | 0.042 | 0.049 | 0.019 | 0.034 |
| Endometrial preparation |  |  |  |  |  |
| HRT | 0.016 | 0.031 | 0.011 | 0.019 | 0.023 |
| NC | 0.029 | 0.058 | 0.041 | 0.051 | 0.034 |
| OI | 0.111 | 0.074 | 0.091 | 0.067 | 0.011 |
| Others | 0.181 | 0.087 | 0.043 | 0.056 | 0.062 |
| Blastocyst quality |  |  |  |  |  |
| High | 1.008 | 0.878 | 0.469 | 0.360 | 0.329 |
| Low | 1.008 | 0.878 | 0.469 | 0.360 | 0.329 |

Values are presented as absolute standardized mean differences (SMDs).

ET, embryo transfer; OPU, oocyte pick-up; BMI, body mass index; AMH, anti-Müllerian hormone; ICSI, intracytoplasmic sperm injection; IVF, in vitro fertilization; HRT, hormone replacement therapy; NC, natural cycle; OI, ovulation induction.
